# Molecular Basis of Calcium-Sensitizing and Desensitizing Mutations of the Human Cardiac Troponin C Regulatory Domain: A Multi-Scale Simulation Study

**DOI:** 10.1371/journal.pcbi.1002777

**Published:** 2012-11-29

**Authors:** Peter Michael Kekenes-Huskey, Steffen Lindert, James Andrew McCammon

**Affiliations:** Department of Pharmacology, Center for Theoretical Biological Physics, National Computational Biomedical Resource and Howard Hughes Medical Institute, University of California San Diego, La Jolla, California, United States of America; Fox Chase Cancer Center, United States of America

## Abstract

Troponin C (TnC) is implicated in the initiation of myocyte contraction via binding of cytosolic 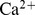 and subsequent recognition of the Troponin I switch peptide. Mutations of the cardiac TnC N-terminal regulatory domain have been shown to alter both calcium binding and myofilament force generation. We have performed molecular dynamics simulations of engineered TnC variants that increase or decrease 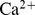 sensitivity, in order to understand the structural basis of their impact on TnC function. We will use the distinction for mutants that are associated with increased 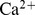 affinity and for those mutants with reduced affinity. Our studies demonstrate that for GOF mutants V44Q and L48Q, the structure of the physiologically-active site II 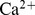 binding site in the 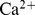 -free (apo) state closely resembled the 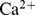 -bound (holo) state. In contrast, site II is very labile for LOF mutants E40A and V79Q in the apo form and bears little resemblance with the holo conformation. We hypothesize that these phenomena contribute to the increased association rate, 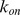, for the GOF mutants relative to LOF. Furthermore, we observe significant positive and negative positional correlations between helices in the GOF holo mutants that are not found in the LOF mutants. We anticipate these correlations may contribute either directly to 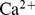 affinity or indirectly through TnI association. Our observations based on the structure and dynamics of mutant TnC provide rationale for binding trends observed in GOF and LOF mutants and will guide the development of inotropic drugs that target TnC.

## Author Summary

Muscle cells contract using a network of thread-like protein assemblies called myofilaments. Contraction is preceded by a signal that causes calcium to rush into the cell cytosol, where it can freely diffuse to and bind the myofilament proteins. Troponin C, a calcium sensor located on the thin filament, initiates and regulates the cascade of changes resulting in the generation of force by the thin and thick filaments comprising the myofilament lattice. In heart tissue, pathological conditions known as dilated and hypertrophic cardiomyopathies (DCM and HCM, respectively) are in part associated with abnormalities in the ability of the myofilaments to generate force at normal calcium concentrations. Manipulation of Troponin C calcium-binding through protein engineering and pharmaceutical intervention has thus attracted considerable attention as a therapeutic strategy for ameliorating these cardiac defects. In this study, we uncover a molecular basis of altered calcium handling for several engineered Troponin C variants, which provides further insight into tuning its control of myofilament contraction.

## Introduction

Sarcomeres contract owing to the translocation of the thick filament, comprised of myosin, along actin chains constituting the thin filament (TF). Contraction is initiated and regulated by Troponin proteins tethered to actin, including Troponin C (TnC), Troponin I (TnI) and Troponin T (TnT), as well as Tropomyosin (Tm). Specifically, 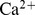 binds to TnC, thereby unveiling a hydrophobic region necessary for binding the TnI switch peptide. Liberation of the TnI regulatory unit from the TF initiates a shift in Tm [1], thus enabling the weak binding of myosin to actin. Subsequent conversion of Tm to the unblocked state permits a cycle of strong myosin binding and propagation along the TF (cross-bridge cycling).

A number of human cardiac diseases including hypertrophic cardiomyopathy (HCM) [2], restrictive cardiomyopathy (RCM) [3] and dilated cardiomyopathy (DCM) [4] have been attributed to mutations in thin filament, thick filament and associated proteins of the sarcomere. RCM and HCM mutations have been shown to increase 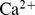 sensitivity of force generation as measured by pCa_50_, whereas DCM mutations reduce this trend. A large number of mutations leading to HCM, RCM, and DCM phenotypes have been collectively identified [5] but only one LOF, DCM-associated mutation has been found in TnC (D75Y [6]). The prominent role of TnC in force development has thus attracted therapeutic strategies to tune its 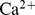 and TnI affinity including drug-design [7] and protein engineering approaches [8]–[10]. In particular, mutation studies of full-length TnC have revealed engineered variants that shift the 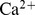 equilibrium constant, 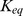 (or pCa_50_), leading to altered force development akin to GOF and LOF [8]. For instance, V44Q and L48Q mutations investigated by [8], [10], [11] have been reported to exhibit GOF-like phenotypes in skinned cardiac fibers, with pCa_50_ values of 6.29 and 6.13 (in isolated F27W TnC), respectively, relative to the wild-type value of 5.48 [8]. Furthermore, Tikunova et al. [10] reported for several GOF mutations including V44Q and L48Q that faster 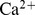 association rates (4.4 to 5.2-fold) contributed more to the increased 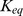 rather than slowed dissociation (approximately 2.8-fold). In comparison, the E40A and V79Q mutations examined by [8] present LOF-like alterations in force generation with pCa_50_ values of 5.16 and 5.30, respectively. While these studies have implicated 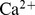 binding as the primary factor in reshaping contractile activity, the structural and dynamical basis of the mutations' effect on TnC is largely unknown.

**Figure 1:**
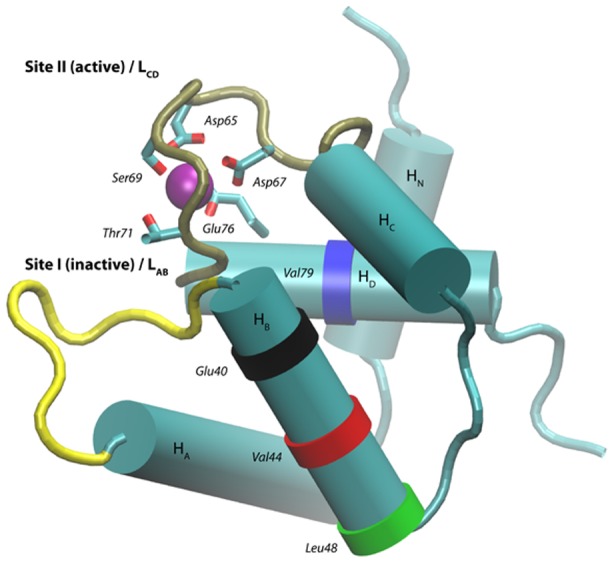
Structure of wild-type cardiac Troponin C. The physiologically *inactive* 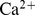 binding site I contains 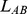 (yellow), while the *active* site II region contains 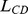 (tan). The locations of the mutants in this study are marked as colored bands, including E40 (black) V44 (red), L48 (green) and V79 (blue).

Structure determination via X-ray crystallography [12] and NMR [13]–[15] has yielded important insight into the molecular basis of TnC function. These studies indicate that TnC consists of two domains: the C-terminal domain is affixed to the thin filament, while the N-terminal regulatory domain is responsible for binding 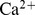 at physiological concentrations, and TnI. The TnC N-terminal domain consists of five helices, HN (4–8), HA (14–24), HB (38–48), HC (54–64) and HD(73–85), two beta sheets 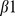 (36–37), 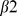 (71–72) (Fig. 1). Loops 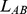 (25–35), 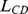 (65–70) of sites I and II form EF hands (helix-loop-helix) that selectively bind 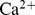, although in cardiac TnC, only site II is physiologically active. Within the EF hand, several acidic residues (D65, D67, and E76) coordinate 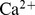, along with S69 and T71; we collectively refer to these amino acids as chelation residues.

Prior experimental and theoretical work have leveraged these structural data to probe rapid, nanosecond timescale conformational dynamics that are correlated with 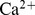 binding [14], [16]. Lim and coworkers [6] characterized a TnC mutant (D75Y) isolated from a patient with DCM and demonstrated its decreased 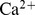 binding capacity and disruption of normal structural dynamics. Varughese and Li further investigated via MD changes in the structural dynamics of cardiac troponin, including TnC, upon binding bepridil, a known inotropic agent [17]. Lindert and coworkers [16] combined long time-scale MD simulations and BD simulations to understand the dynamics of wild-type TnC in its 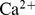 -free, 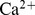 -bound, and TnI -bound states, as well as V44Q [18]. Recently, a combined experimental and theoretical approach examined 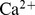 binding and the structural stability of a GOF mutant L48Q [19].

We seek to extend these studies by 1) comparing LOF and GOF mutants to better contrast differences in apparent 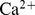 sensitivity and 2) explore longer simulation times comparable to dynamics captured by NMR order parameters. By a combination of molecular and Brownian dynamics, our approach identifies structural and dynamic factors impacting 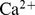 binding in light of GOF and LOF mutations. The outcome of this study provides greater insight into the mechanisms of structure/function relationships for N-terminal cardiac TnC that are important to myofilament activation.

## Results

### Mutations slightly disrupt wild-type TnC structure

#### Overall TnC structure

**Figure 2:**
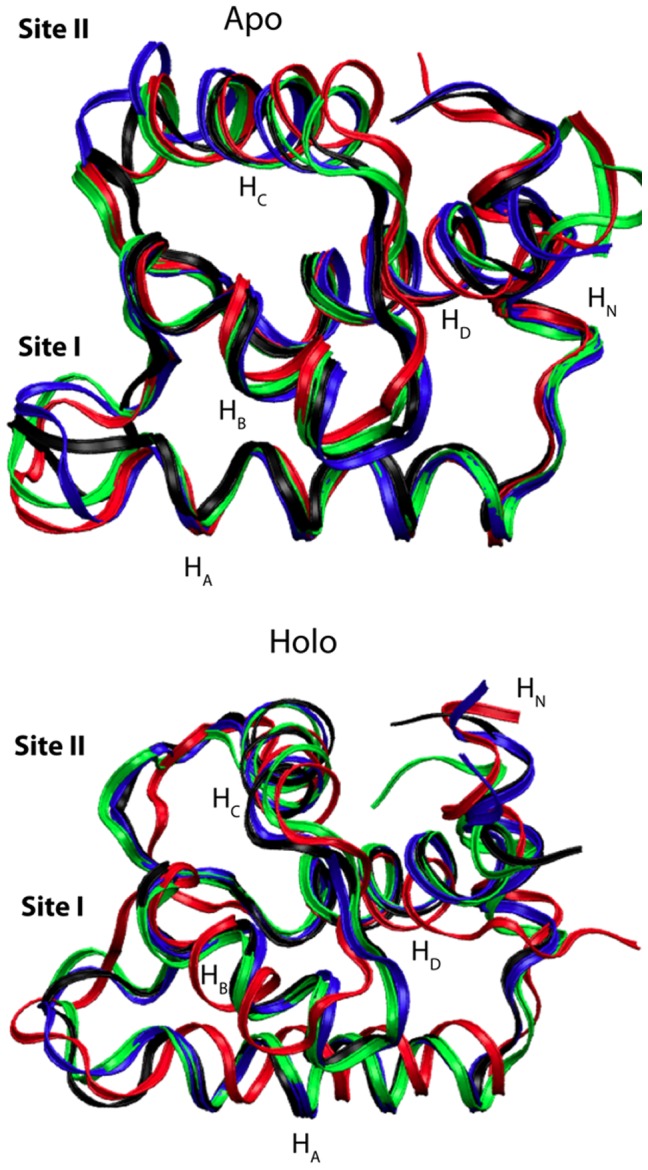
Superposition of representative molecular dynamics snapshots for apo and holo mutant structures. apo and holo states demonstrate the similar backbone structure for E40A (black) V44Q (red), L48Q (green), and V79Q (blue).

To understand the impact of GOF- and LOF- associated mutations on the structure of the N-terminal regulatory domain of TnC and subsequent impact on 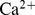 affinity, we performed *in silico* mutations of the 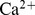 -bound (holo) and 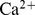 - free (apo) wild-type NMR structures (1AP4 and 1SPY). The mutations included the GOF-like mutants V44Q and L48Q, as well as the LOF-like mutants V79Q and E40A. These mutants induced significant changes in the polar character of wild-type TnC, with V44Q, L48Q, and V79Q representing apolar to polar alterations and E40A, a charged to neutral substitution. It is plausible that these mutations might disrupt the hydrophobic packing of the wild-type TnC structure [8]. Our simulations indicated that the mutations preserved the overall arrangement of helices as evidenced by the superimposed structures in Fig. 2 after equilibration, with relatively minor 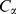 RMSD differences with respect to wild-type (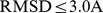) (Fig. S2a). While the 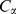 RMSD does not strongly distinguish between GOF and LOF mutants, differences were noted at 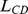, the physiologically active 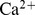 binding domain, with increasing deviations noted for V44Q, E40A, V79Q, and L48Q (RMSD from 1.0 to 3.5 Å, (Fig. S2c). Differences between structures were comparatively less apparent for all other helices and loops. An exception to this trend was noted for V44Q, which displayed significant RMSD changes for 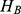, 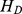 and 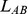. In the subsequent results and discussion, we will argue that these deviations likely corresponded to low-frequency, collective motions involved in TnI recognition.

#### Local conformation and electrostatic potential

With such similar backbone configurations noted amongst the mutants, we examined whether changes in electrostatic potential, particularly near the mutation sites, could account for altered 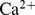 association rates. In the wild-type structure, the native residues at the apolar mutation sites in wild-type TnC were well-buried, while E40 was solvent-exposed. The side chains of the glutamine mutations consistently rotated toward the solvent, with minor changes in 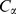 positions (data not shown). Furthermore, all glutamine mutations except V79Q appeared to freely rotate and presented only transient interactions with neighboring residues. For V79Q, the mutated side chain was found to intermittently interact with the HN backbone, but generally remained free. For E40A, the mutation of the solvent-exposed glutamic acid left a small, solvent-exposed apolar patch. Adaptive Poisson Boltzmann Solver calculations indicated that the mutations induced localized changes in the electrostatic potential (Fig. S4). However, the electrostatic potential near site II in the apo form, where 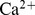 associates, and near the TnI binding region in the holo form, were quite similar, even amongst geometrically-diverse clustered snapshots from the corresponding trajectories.

#### Calcium association

The collision of freely diffusing 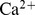 with TnC constitutes the first stage of the 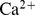 association rate (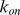). To further probe whether subtle changes in the electrostatic potential impacted the collision frequency, otherwise known as the diffusional encounter rate, 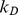, we computed 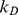, for several conformations of each mutant from the MD simulations. We found that the diffusional encounter rate was practically indistinguishable amongst mutation types (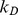 = 5.7
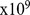
–2.9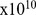 [1/Ms], Table 1). These results were not unexpected, as 1) the general shape of the apo TnC structures were remarkably similar (Fig. 2) and 2) the electrostatic potential (as computed by APBS) was negligibly different near the 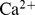 binding domain (Fig. S4).

**Table 1:** Ca2+ association rates.

cTnC	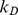 [1/Ms]
E40A	5.7 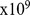
L48Q	1.1 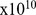
V44Q	2.6 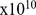
V79Q	2.9 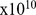

Predictions based on Brownian dynamics.

**Figure 3:**
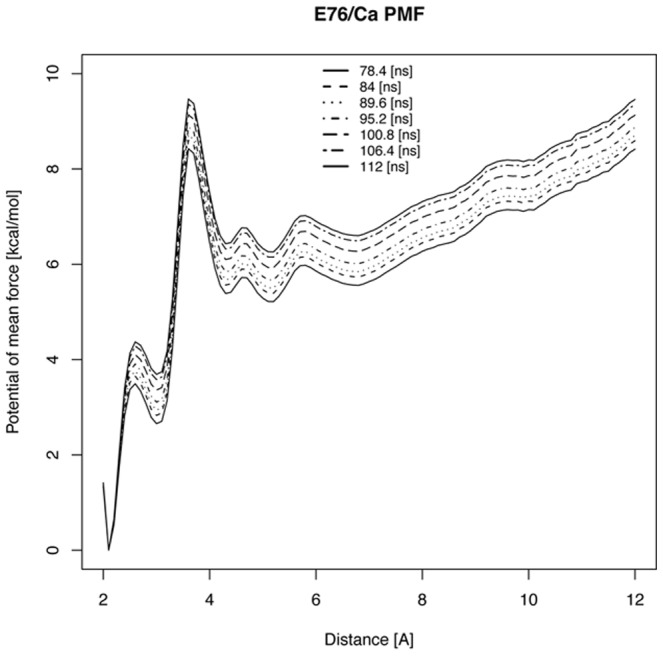
Calculated potential of mean force for Ca2+ approaching wild-type cardiac Troponin C site II binding region. Potential of mean force [kcal/mol] is measured by distance between 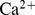 and C*_γ_* of D76.

Following the diffusional encounter between 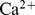 and TnC, we speculated that the protein undergoes a slight conformational reorganization to fully coordinate 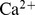 in the bound state; the rate for this process may be combined with 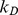 to give the overall association rate, 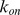. We used the ABF [20] method in NAMD to determine the free energy barrier separating the encounter and bound states, by computing the PMF along a reaction coordinate representing the distance from the bulk solvent toward E76 of site II for the apo wild-type structure from [16] (Fig. 3). We found that the PMF decreased as the 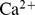 ion approached site II with exception to a roughly 3 kcal/mol barrier at approximately 4.0 Å from the binding site. Examination of the ABF trajectories suggests that the largest barrier arose from a change in D67 coordination of 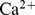 (bi-dentate to mono-dentate), mono-dentate coordination via E76 (Video S 1), and reorganization of the five waters coordinating 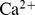.

### Dynamics of protein domains

A requisite for high affinity binding is competent formation of 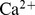 site II interactions. To gauge the integrity of this 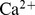 coordination amongst the mutants, we report the mean residue/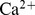 distance for site II residues D65, D67, S69, T71 and E76 in Table 2 and Fig. S3. We found close coordination (distance 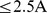) of 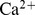 with D65, D67 and E76 (Fig. S3) that evidence strong 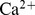 binding and were consistently preserved over the simulation period. We classified the interactions with 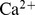 as mono-dentate versus bi-dentate interactions and found that E65 is primarily mono-dentate, E76 is bidentate, and D67 oscillated between mono- and bi-dentate coordination. S69 and T71 exhibited larger distances of approximately 4 to 5 Å (not shown) indicative of comparatively weaker interactions. Altogether, chelation distances were consistent and well-stabilized across holo cases, with no distinction between LOF and GOF mutants.

**Table 2:** Summary of 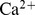/Site II residue distances [Å] for mutants and wild type TnC.

Resid/Atom	Wild-type	V44Q	L48Q	E40A	V79Q
65 OD1	2.26	2.55	3.33	2.88	2.11
65 OD2	2.96	3.41	2.74	2.79	4.13
67 OD1	2.69	2.69	2.84	2.30	4.21
67 OD2	3.24	2.17	2.18	2.21	2.12
69 OG	5.95	4.95	5.48	4.68	5.99
71 OG	2.77	3.79	4.22	4.19	4.30
71 OG1	4.56	5.58	5.97	5.87	5.70
76 OE1	2.14	2.23	2.23	2.23	2.20
76 OE2	2.72	2.21	2.21	2.21	2.19

#### Order parameters

**Figure 4:**
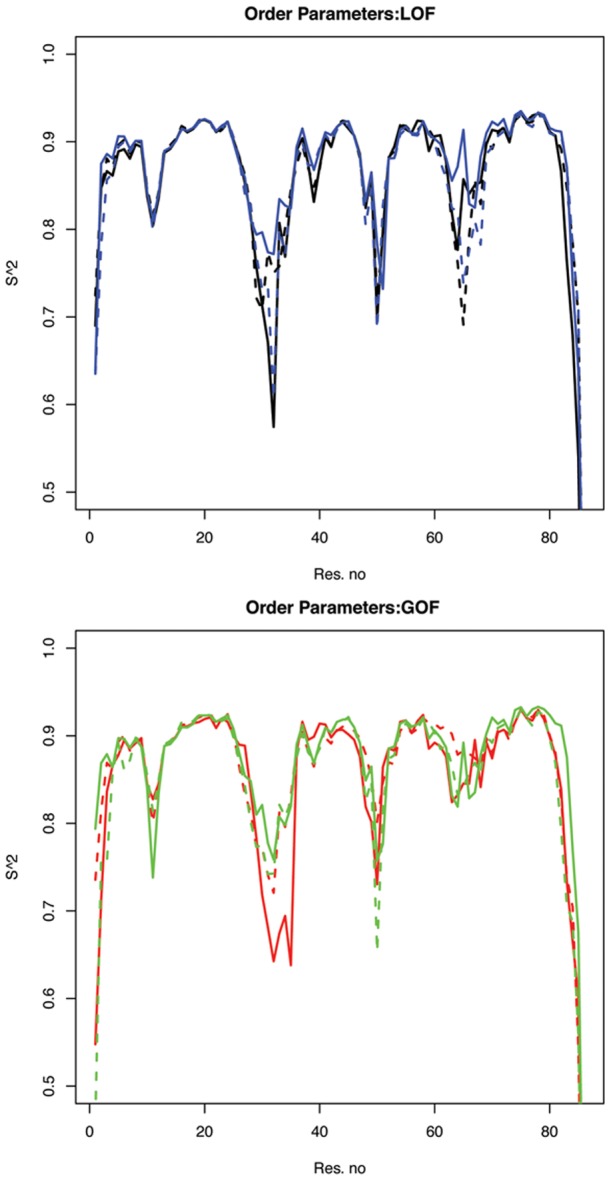
Simulated 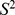 NMR order parameters. Order parameters are provided for apo (dashed) and holo (solid) for LOF mutants V79Q (blue), E40A (black) b) and GOF mutants V44Q (red), L48Q (green). Site I and site II are located at approximately residues 28–38 and 67–76, respectively. revN:19.

Our prior simulations with wild-type TnC indicated a significant decrease in site II dynamics upon 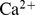 binding [16]. Since the mutants in this study impact the ability to bind 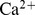, we postulated that the mutations may tune the conformational dynamics of site II. To this end, we report computational estimates of NMR order parameters for the backbone amides, as well as correlations between residue displacements. Our computational estimates of 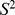 based on the N-H bond vector in Fig. 4 reflected the relative flexibility of the protein backbone, with 0 and 1 representing very dynamic and static NH bond vectors respectively). Our results evidenced smaller order parameters, and hence larger dynamics, at site I and II relative to the baseline (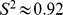) for all mutants, consistent with trends observed for the wild-type [16]. Binding of 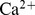 in the holo state stabilized site II in particular, and yielded similarly increased order parameters (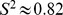) for all mutants. We furthermore noted for both sets of mutants a region of increased dynamics near residue 50 of 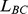 (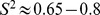), that was not present in the wild-type. We attributed this destabilization to a disruption of the hydrophobic packing between  and 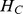 induced by both GOF and LOF mutations. 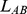 order parameters were generally comparable amongst the mutants for the apo form (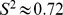), with exception to V79Q with 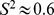. This region varied considerably in the holo form, with no clear distinction between GOF and LOF.

In the apo state, we report clear trends discriminating GOF and LOF mutants at 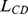 comprising site II. For LOF mutants, the apo 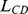 order parameters are nearly comparable in magnitude to those at 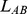, suggesting a comparable degree of dynamics (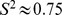 versus 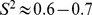). In contrast, 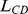 for GOF mutants was *considerably less dynamic in the apo form* (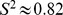) and 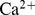 binding practically had no effect on the order parameters. This striking difference in  order parameters between 1) the apo and holo states and 2) the LOF versus GOF mutants further illustrates the important role of site II dynamics in 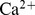 binding, with lesser disorder in the apo state potentially reducing a loss of entropy upon binding 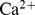.

**Figure 5:**
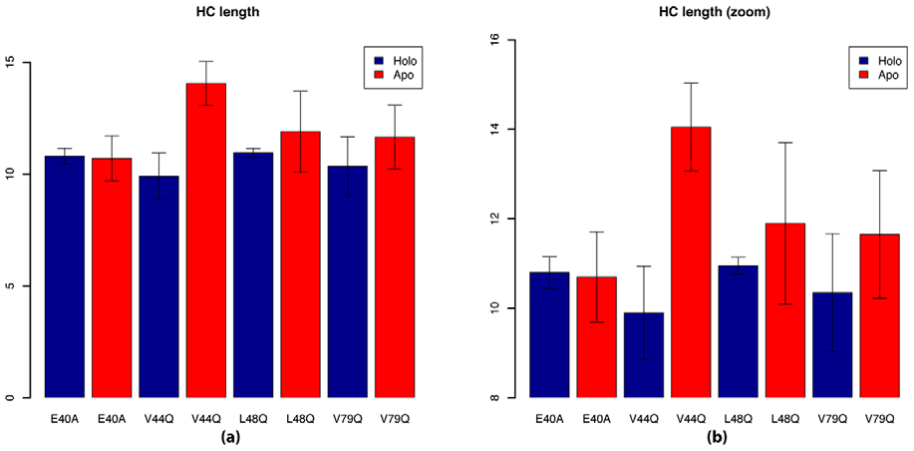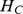 helical lengths. a) Helical lengths based on the entire MD simulation for apo structure are reported in red, and holo structure in blue. b) scaled representation of a).

To understand the structural basis for the differences in 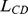 dynamics amongst the apo structures, we examined the helical content of 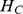 by counting the number of alpha helical residues between P54 and G68. Differences in 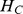 helical content were most apparent for V44Q compared to E40A (14.1 versus 10.7), yet the 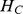 length was only marginally greater for L48Q relative to V79Q (11.9 versus 11.7)(Fig. 5). Upon binding 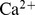, 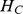 helical length was found to consistently decrease, representing a partial unfolding of the helix.

**Figure 6:**
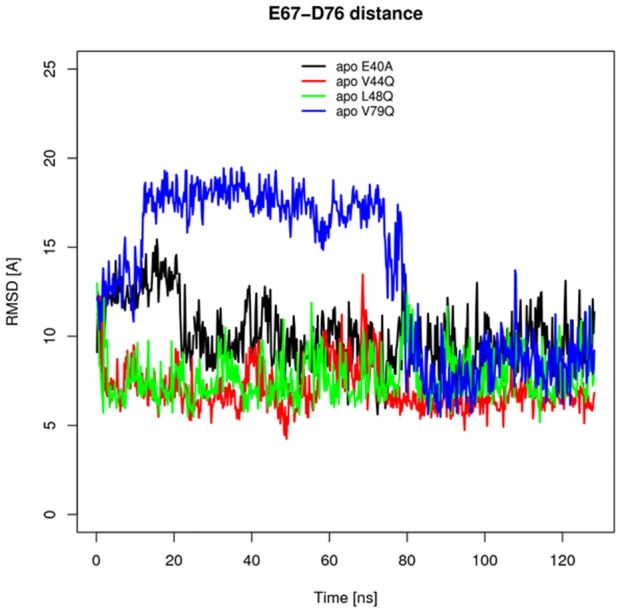
Distance between D67 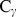 and E76 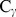 of the apo-state structures. Distances are reported in [Å] for E40A (black) V44Q (red), L48Q (green), and V79Q (blue).

To examine the consequences of increased 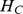 length on 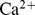 binding, we compared the distances between D67 (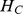) and E76 (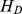), which coordinated 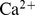 in our PMF calculations (Fig. 6). We found for the GOF apo mutants that the 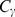
s of the polar residues approached 6 Å, which is considerably smaller than average values from simulations of the wild-type structure (10.8 Å, unpublished). In contrast, the distances were much larger for the mutants, to the extent that these residues were essentially non-interacting. This was indeed the case, even for V79Q, whose 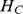 length was comparable to mutant L48Q. It appeared that the close pairing between D67 and E76 was made possible by a transient network of hydrogen bonds between solvent molecules and the carboxylic acid side chains. In contrast, the D67 side chain for the LOF mutants was rotated outward toward the solvent, either through slight displacement of 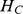 relative to 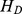 (V79Q), or unwinding of 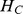 (E40A). We further demonstrate in Fig. S1 that the range of D67/E76 distances was more compact relative to those reported by the LOF cases. We anticipate that this D67/E76 interaction may contribute to the increased stability of the 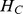 helix for at least the V44Q mutant. Moreover, the tight D67/E76 pairing may further constrain 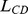 to a holo-like conformation, thus facilitating the 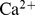 binding event depicted in the supplemental movie (Video S 1).

#### Correlations

**Figure 7:**
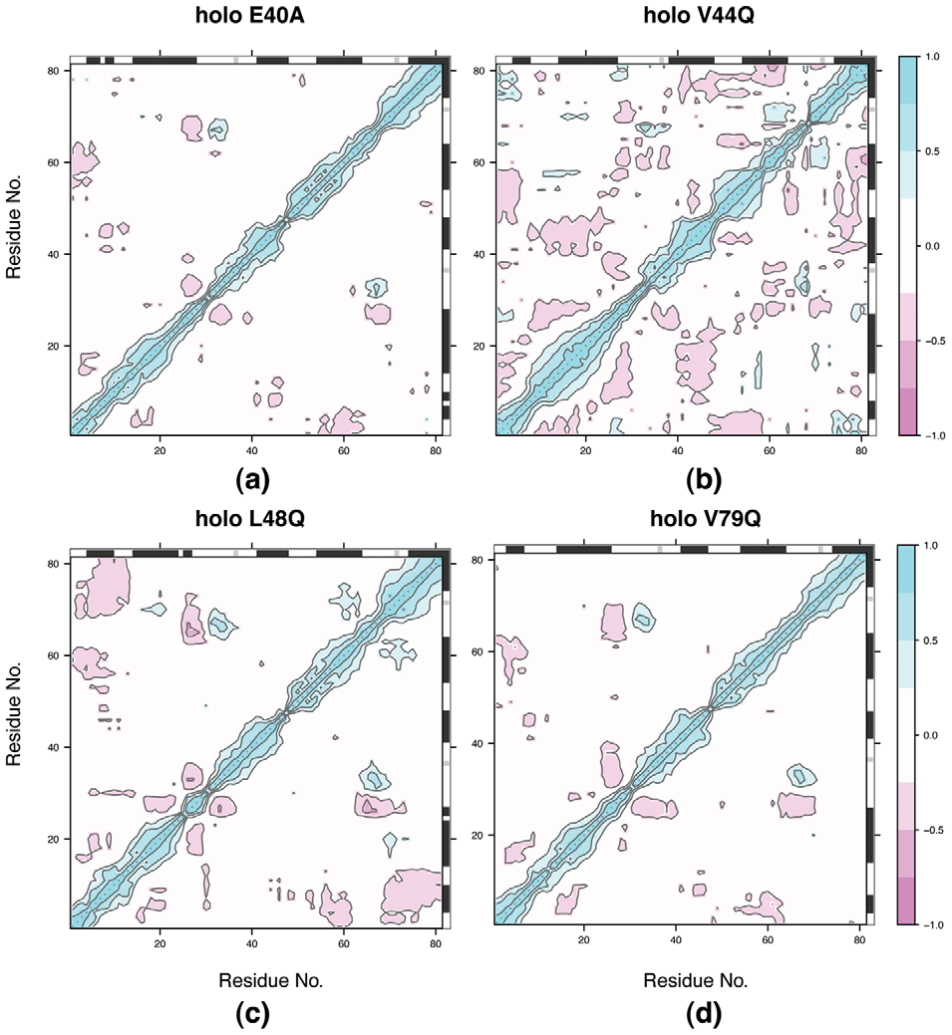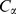 correlations for holo TnC mutants. a) E40A b) V44Q c) L48Q d) V79Q. Positive correlations are reported in blue, negative correlations in red. Approximate secondary structure displayed along axes, with 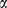 helices marked in black and 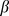 sheets in gray.

While the GOF and LOF holo cases presented similar order parameters, we turned to residue-based correlations of the holo state to provide additional insight into 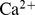 and subsequent TnI binding (Fig. 7 and Table 3). Common to all mutants and binding states were substantial correlations between 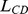 and 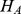 (negative) and 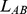 (positive). Moreover, we observed a ‘cross’ pattern centered about I26 of 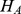 that arose from negative correlations between 1) the C-terminal 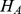 segment and the entire 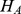 helix, and 2) the 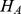 segment with 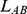. We speculate that these correlations, which appeared to be more extensive in the GOF mutants relative to LOF, may alter the 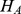/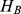 angle, which is associated with TnI recognition. Negative correlations between 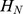 and 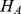 or 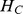 were also observed, which correspond to the TnI binding region. Interestingly, we also observed extensive, off-diagonal negative correlations in the holo GOF mutants that were diminished in the LOF data. Among these, we consistently found negative correlations between 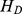 and 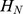 (L48Q) or 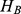/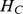 (V44Q), as well as positive correlations between 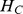 and loop 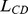 of site II common to both. Similar correlations involving 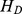 were also observed for the wild-type (see Fig. 6C, [16]), but not in the LOF mutants. Moreover, the prominent 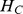 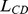 correlations appeared to be exclusive to GOF mutants. While these correlated motions did not lead to any obvious large-scale conformational changes, their prevalence in GOF mutants and to a lesser degree, the wild-type, appeared to loosely follow the trend of enhanced binding affinity.

**Table 3:** Summary of correlations in TnC mutants.

Case	Mode	V44Q	L48Q	(E40A/V79Q)
All	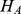 , 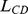	−	−	−
All	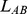 , 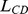	+	+	+
All	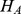 , C-terminal 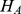	−	−	>0
All	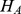 , 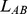	−	−	−
All	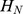 , 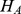 / 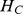	−	−	−
Holo	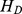 , 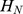	+	−	0
Holo	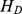 , 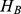 – 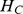	−	0	0
Holo	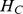 , 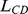	+	+	0

+ designates increase, - decrease, and 0 no change.

#### 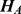 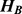 angles

**Figure 8:**
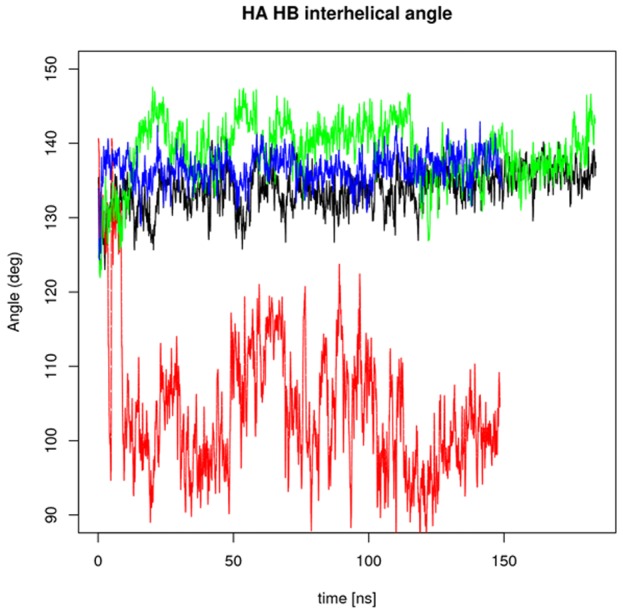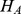 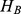 interhelical angle for the holo state. Angles reported in [deg] for E40A (black) V44Q (red), L48Q (green), and V79Q (blue) package.

Motivated by the observation of correlations in regions associated with TnI recognition, we examined whether these motions could contribute to an opening event that exposes the TnI binding region of TnC. Oleszczuk et al previously demonstrated that upon binding TnI, cardiac TnC undergoes a closed to open conformational transition corresponding to movement of helices 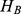 and 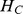 away from helices 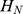, 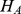, and 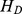, which were characterized by measuring chemical shifts for L29, A31, K39 and E66 [21]. This transition is observed in the 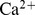 and TnI -bound states of skeletal TnC [22], but only in the TnI -bound form for cardiac TnC ; it is accompanied by a decrease in the 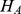/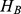 angle from 140 and 132 degrees (where the helices are nearly parallel) for the apo and 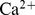 -bound structures (PDB codes 1SPY/1AP4) to 102 to 121 degrees (where the helices approach perpendicularity) for various TnI -bound states (PDB codes 1MXL, 1J1E, 1LXF). Thus, we measured the 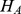 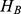 angle in Table 4 and Fig. 8. We found in fact that V44Q holo state assumed 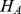 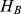 angles in the 100–110 degree range, indicative of potential opening events. In Fig. S5 we have overlaid this structure with the wild-type TnC in the presence of 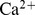 and TnI to demonstrate that the opening event led to exposure of the TnI -binding pocket. All other species reported angles between 135–140 degrees, with L48Q slightly closing during the simulation (represented as increase in 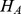 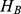 angle).

**Table 4:** Interhelical angle.

state	V44Q	L48Q	E40A	V79Q
apo	134	132	134	129
holo	104	138	134	136

Average 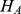 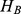 interhelical angle [deg].

## Discussion

### Summary of structural differences

Our simulations of GOF and LOF variants of the TnC N-terminal regulatory domain recapitulate a number of structural predictions in [8] based on mutants of the full-length F27W TnC For instance, differences in alpha helical character in the apo state were found amongst all mutants, particularly at 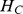, with V44Q and E40 presenting the largest increase and decrease, respectively, while V79Q and L48Q had intermediate values. These findings mirror those reported using circular dichroism [8], although in this study, the measurements assessed the alpha helical content of the *entire* protein. We also observed a decrease in 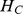 length upon 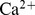 binding, yet Parvatiyar et al. report an increase in helical secondary structure for the full-length TnC. Since our simulations focus solely on the N-terminal domain, it is possible that 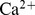 binding might increase helical content of the C-terminal domain or linker region, thus offsetting decreases at 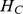.

Moreover, we discovered that the packing of TnC helices was disturbed for almost all of the mutants we studied. For instance, among the GOF mutants, the V44Q mutation significantly disrupted the hydrophobic contacts between helices 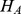 and 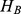 in the holo state (Fig. S6a), as was originally proposed by [8] and [10]. This destabilization may contribute to the larger 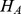 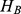 opening angle and frequency for this mutant. L48Q, on the other hand, presented slight structural changes involving 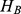 that evidence disruption of the hydrophobic packing, but nevertheless did not cause noticeable 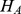 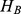 opening in our simulations (Fig. S6b).

For the LOF mutants, in remarkable agreement with Parvatiyar [8], we found that the V79Q mutation formed a possible hydrogen bond with D75, a mutation site involved in (D75Y [6]) (Fig. S6c). This alteration in the side chain conformation appeared to disrupt the packing around 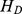, which may reduce the ability to form the close D67/E76 contacts observed for the and wild-type cases. In contrast, we found that the E40A mutation did not disrupt the packing, per se, but rather decoupled the WT's apparent hydrogen bond network linking S37, E40 and R43 (Fig. S6d). Interestingly, its impact appeared to be largely allosteric in nature, as we observed an increase in site II dynamics despite a minimal change at sites 37 and 43. One explanation for this allosteric change is that the mutation alters the coupling of beta sheets bridging sites I and II, thus perturbing the 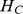 length and D67/E76 pairing. While these changes amongst mutants are quite apparent from relatively short MD simulations, elucidation of longer timescale conformational changes through direct simulation or extrapolation of principle components [16] may provide additional insight into the role of TnC during the comparatively slow process of sarcomere contraction.

### Thermodynamic factors contribute to differences in 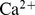 affinity among TnC mutants

In this study, we sought to understand why 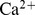 affinity is enhanced in isolated TnC for GOF mutants and decreased for LOF mutants. If the mutations predominantly impacted the association rate without affecting the equilibrium constant, one might speculate that the mutations alter the transition state barrier to 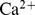 binding. However, the equilibrium constants measured in [10] were in fact found to substantially vary, thus we sought to identify qualitative factors contributing to the free energy of the apo and holo states. We examined this by considering structural and dynamic factors for both apo and 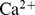 bound TnC mutants. Firstly, the binding free energy is determined by the difference between the free energy of the bound substrate-receptor complex versus the isolated apo-receptor and substrate. Our initial hypothesis was that and mutations may either disrupt the native structure of TnC or alter the ability of TnC to coordinate 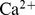; however, we found that overall the apo and holo states share remarkable structural similarity, based on RMSD comparison of the entire N-domain. with localized disruptions in the packing about 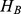 and near 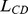, where 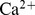 binds.

Second, we evaluated the magnitude of the electrostatic interactions between substrate and receptor as a determinant of ligand binding affinity. Because of the structural similarity amongst the mutants and the remote position of the mutations relative to site II, we did not observe any appreciable changes in the electrostatic potential near the binding site. This is in contrast to the case of D75Y explored by Lim et al., for which the charged-to-neutral mutation within site II would plausibly cause a significant change in the local electrostatic potential [6]. Instead, we found that strong electrostatic interactions between the 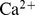 ion and its chelating residues afforded tight coordination of 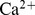 as evidenced by the constrained dynamics in 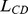, despite minor changes in RMSD amongst the mutants. This demonstrates that TnC is tolerant of changes in backbone conformation, provided that the chelation residues can form optimal interactions with 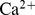. This is not surprising, as the highly negatively charged chelation residues are strongly attracted to the divalent cation, and thus the driving force for forming the optimal conformation is likely quite large. In so far that the enthalpy of binding is dominated by the charge-charge interactions arising from 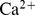 binding, it is plausible that the enthalpic contribution to binding is roughly comparable amongst the mutants. Estimating this contribution is the subject of a follow-up investigation.

Given the qualitative similarity of this electrostatic enthalpic contribution, it is therefore possible that entropic considerations are more significant for discriminating low- from high-affinity mutants, based on our estimated order parameters and correlation data. Common to both mutations types are dynamic regions at sites I, II and 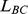 as evidenced by our estimates of NMR order parameters. However, a key distinction between the mutation types is that the site II binding domain in the apo state is more rigid, and practically identical to the holo state, for the GOF mutants, in sharp contrast to the LOF mutants and findings for WT TnC (Fig. 8B [16]). In so far that order parameters are a qualitative measure of entropy [23]–[25], a lesser change in order parameters for the GOF relative to LOF mutants in this study could reflect a smaller entropic cost for binding 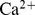 and thus result in increased affinity. These findings indicate that changes in 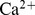 affinity are driven by alterations in TnC dynamics, not in 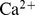 coordination, in agreement with [8]. They raise the possibility that calcium sensitivity modulation of TnC might be controlled through control of 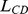 dynamics.

Common to both species are also extensive correlated motions in the apo states, as well as a conserved set of correlation patterns in the bound state. GOF and LOF species both exhibited significant anti-correlation between 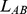 and the C-terminal end of 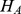 near I26. However, only for GOF mutants did we observe a complementary anti-correlation between the C-terminal region of 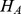 and the entire 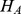 helix. We postulate that this motion alters the 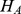 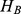 angle, for which we speculate that I26 serves as a pivot point though its hydrophobic interactions with 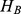. We observed additional correlations between helices 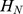 and 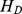 for GOF and wild-type (see Fig. 6C, [16]), but not for LOF mutants, which indicated the V79Q and E40A mutants may have destabilized the hydrophobic residues between these helices in line with previous predictions [8], [19]. It is further possible that the greater extent of positive correlation between 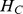 and 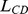 for the GOF cases relative to the wild-type indicates more extensive stabilization of site II and perhaps contributes to their enhanced 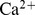 affinity. Similarly, we saw a greater number of off-diagonal correlations for GOF relative to LOF, which may evidence greater entropic stabilization of the entire protein for the GOF cases. In comparison to the wild-type correlations reported in Lindert et al., the comparable off-diagonal correlations for L48Q and considerably more extensive motions for V44Q are in line with the latter having greater 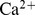 affinity. Therefore, in addition to the enthalpic factors stated in the previous section, 1) GOF mutants may have a more favorable, entropy-stabilized bound state relative to LOF, 2) the distribution of correlated motions may play a role in increasing 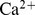 affinity.

### Diffusion and post-encounter factors affect 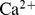 association rate

Interestingly, two mutations (L48Q and V44Q) were reported to have a larger impact on 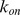 versus 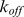, with 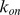 four- to five-fold larger with respect to wild-type relative to a partially compensating three-fold increase in 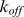 [10]. A secondary objective of this study was thus to shed light on the molecular basis of altered 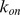
s for the mutants, and to determine whether complementary mechanisms were at play for the cases. The diffusional component of the association rate, 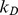 is strongly influenced by electrostatic interactions and geometry, thus we sought to determine to what extent these factors impacted 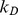. To explain our results, we resort to the the transient-encounter complex theory formulated by Zhou and others [26], which suggests that association is divided into two regimes - diffusion limited binding to the protein surface (transient encounter complex), followed by a post-encounter reorganization. The transient encounter complex can be defined as the surface at the cusp of the bound and unbound potential energy surfaces, whereas the post-encounter reorganization corresponds to localized conformational changes that bring the transient complex to the native bound state.

Electrostatics are known to often drive association between substrate and receptor to form the encounter complex. Our predictions of 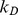 were on the order of 10^9^ to 10^10^ [1/Ms], which suggests the prominent role of electrostatics in driving the initial states of 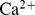 association, as found for the wild-type [16]. However, since the electrostatic potential and predicted diffusional encounter rates were nearly indistinguishable amongst the GOF and LOF mutants, differences in the experimentally measured 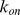 values were not likely electrostatics in nature. This leaves the post-encounter regime as a possible discriminator amongst the mutants. Furthermore, as we later argue, post-encounter events may reconcile the discrepancy between the magnitude of the predicted 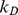 s and the substantially smaller estimates for the *overall* 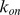 (10^7^ to 10^8^ [1/Ms] [27], [28] for the wild-type.

When 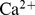 reaches site II we postulate that several post-encounter factors impact the rate of binding, including enthalpy and the reorganization time associated with an induced-fit mechanism. Based on the highly solvent-exposed binding site and abundance of negatively charged residues that coordinate cations, we expected a highly favorable enthalpic interaction as 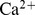 approached site II along the reaction coordinate. However, coordination of 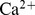 required the energetically-unfavorable displacement of the solvation shell around 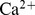 ; this partial dehydration appeared to be accompanied by poly-dentate interactions with D67 and E76. Our PMF calculations indicated that these offsetting energetic factors manifest a modest barrier of 3 [kcal/mol], which we expected would reduce the association rate by nearly three orders of magnitude (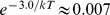 by Kramer's theory). This explanation could align our predicted 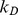 values of 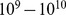 [1/Ms] with experimentally-observed 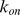
s (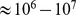 [1/Ms]). Since the coordination number of 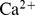 is identical across all mutants, we speculate that the energetic cost of 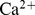 dehydration should be similar for all cases; if dehydration in fact dominates the free energy barrier, we might expect a comparable decrease in association rates amongst the studied mutants.

The differences in the time required for the induced fit of 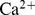 from the apo state are expected to impact the overall association rate. Our simulations of the apo state indicated that site II more closely resembled the holo state for GOF relative LOF to mutants, which we attributed to tighter D67/E76 interactions. The close D67/E76 distance likely contributes to, or benefits from, the increased 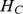 character of V44Q. While the 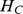 helical length for L48Q is marginally greater than the LOF mutants and wild-type (data not shown), it is apparently sufficiently stable to ensure D67/E76 pairing. We speculate that the close pairing of D67 and E76, through transient coordination of waters near site II, may contribute to optimal placing of 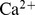 chelation residues and thus constitute a ‘pre-formed’ binding site. Because the GOF mutants appear to spend more time in productive conformations relative to LOF mutants (as measured by D67-E76 distance), the reduced time required to form the binding site could account for their faster experimentally-observed 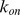
s. This model could explain the findings of Tikunova et al. [10], who suggested that mutations shift equilibrium between bound-form and apo form, thereby giving rise to enhanced binding affinity for the GOF mutants.

### Structural dynamics contributing to Troponin I recognition

In the previous section, we rationalized trends in altered 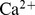 affinity based on apo and 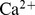 -bound TnC, in order to explain experimental results obtained in *absence* of TnI. In physiological systems, however, TnI binding is known to enhance 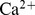 affinity for TnC, primarily because of the activation of cross-bridges and subsequent alteration of myofilament lattice properties during rigor [29]. In this context, it is possible that the correlated motions observed in holo TnC mutants promote productive TnI binding, although this was not explicitly modeled in our study. Our future work aims to include the contribution of the myofilament lattice to 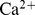 association, at which point more direct comparisons against cooperativity data can be made.

Nevertheless, a prerequisite for the formation of the open state supporting TnI binding is the exposure of a hydrophobic patch between residues L29, A31, K39, and E66 [21]. The transition to the open state entails the progression of helices 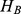/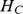 (HB) away from helices 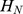, 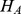 and 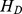 (NAD), which is associated with enhanced 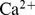 affinity [10] and is suggested to be a common mechanism for the enhanced 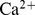 affinity observed for several mutations along the BC/NAD interface [30]. Our simulation results show substantial exposure of this region for holo V44Q mutant, as evidenced by 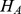/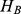 angles approaching 100 degrees, versus 130–140 degrees typically observed for the closed apo and holo states of wild-type cardiac TnC. Specifically, for V44Q we observe that 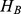 significantly deviates from the TnC NAD core, which would be in line with its greater affinity in the intact thin filament. For all other mutants, including the L48Q GOF mutant and the WT (see Figure 2C in [16]), 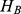 movement is minimal and only transient, sub-nanosecond decreases in the 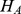/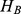 angle to the 100 degree range are observed, which are likely too fast to be resolved given the temporal resolution of NMR. Thus, either 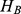 movement is slow relative to our simulation timescale or more likely, TnI plays an active role in stabilizing the open state. In fact, we have recently observed in micro-second timescale simulations of TnC that the V44Q mutation reduces the free energy barrier to decreasing the 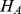/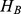 angle, which may increase the rate of forming the open state [18].

We now focus on correlated motions that may contribute to an opening event. Prior studies [6] suggest that concerted motions might be implicated in 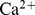 affinity, either directly or indirectly through enhanced interactions with TnI. One speculation based on our correlation data is that the positive correlation between beta sheets 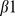 and 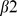 (of 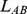 and 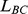) initiates a signal between 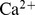 binding at site II and the exposure of the TnI binding surface. We base this on the correlation/anti-correlation pattern between 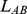 and 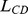, reflect tugging of 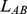 away from the 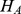 terminus. By tugging on the 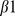, which is directly adjacent to the base of 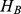, we would expect a widening between 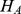 and 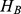 that exposes the hydrophobic surface for TnI. The strong correlation between 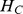 and 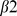 may amplify the effect of the tugging between loops and increase the propensity of 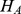 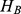 widening in mutants.

### Conclusions

We have identified trends that readily discriminate between GOF and LOF mutants. Our findings suggest that differences in calcium sensitivity cannot easily be explained in terms of large structural changes or differences in the electrostatic potential. Instead, the modulation of calcium binding may be due to dynamic motions (fluctuations and correlations), as well as the time associated with reorganizing the binding site for optimal 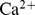 - TnC interactions. These findings suggest that modulation of the *dynamic* properties of TnC via mutation may represent an attractive avenue for tuning myofilament contraction. At the same time, theoretical investigations aiming to characterize the thermodynamics of 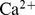 binding from simulation would benefit from considering the contribution of multiple, highly dynamic conformational states in equilibrium. We are currently examining whether these findings are reflected in the -associated TnC mutant D75Y. Moreover, to examine the molecular basis of impairment myosin ATPase inhibition characteristic of and observed for the V44Q and L48Q mutants [8], we seek to apply our modeling approaches to a model of the intact myofilament [12].

## Materials and Methods

### Structure preparation and simulation


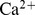
-bound (1AP4) and 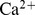-free (1SPY) NMR structures resolved by [14] were obtained from the Protein Data bank [31]. E40A, V44Q, L48Q, V79Q mutations were performed using the Mutate Residue module in VMD [32]. VMD was used to add a TIP4P water box with a 20 Å boundary on the protein as well as sufficient KCl to obtain charge neutrality and a 0.15 M solution. Protein and TIP4P waters were parameterized using the Charmm27 [33] force field. Mutated side chains were subjected to 1000 steps of conjugate gradient minimization with the remainder of the protein held fixed. Using NAMD 2.7b [34], the solvent was equilibrated for 5000 steps of NVT MD protein-fixed simulation using a 2 fs integration step at T = 310 K. 12.0 Å cutoffs were used for non-bond terms, and PME tolerance, interpolation order and grid spacing were set to 10e-6, 4, and 1.0 Å ‘respectively. Harmonic constraints of 10 [kcal/mol] were placed on the protein, which was equilibrated for an additional 5000 NVT steps. 5000 steps of unconstrained NVT, followed by 30000 steps of NPT MD were used for the final equilibration steps. Production runs using the NVT ensemble were at least 120 ns in duration.

### Potential of mean force calculations

The Adaptive Biasing Force protocol [35] in NAMD was used to estimate the PMF. Structures and MD parameters follow from the the previous section. The colvars procedure in NAMD was used to guide the atom along the reaction coordinate, here chosen as the distance between 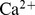 and the E76 carboxylic acid through the definition of the AtomDistance colvar group. Lower and upper boundaries of 2 and 12 Å, varied in 0.1 Å increments were chosen for the colvar AtomDistance, while upper and lower wall constants were both set to 10 [kcal/mol]. Colvar statistics were collected at 4 ps intervals and the *fullSamples* parameter was set to collect 500 samples at each window prior to biasing. 11 ns of ABF simulation were performed for the PMF estimation. Simulation files for NAMD are provided at Video S 1

### Analysis of molecular dynamics trajectories

Trajectories were analyzed using the R statistical package Bio3D [36]. Pearson correlation coefficients were computed using the Dynamic Cross Correlation Maps (dccm) package implemented in Bio3D. Molecular dynamics snapshots (taken every 6 ps from the trajectories) were aligned by the protein's C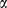 atoms and subsequently clustered by RMSD using GROMOS++ conformational clustering [37]. A RMSD cutoff of 1.5 Å (apo E40A), 1.8 Å (apo L48Q), 1.7 Å (apo V44Q, apo V79Q), 1.6 Å (holo E40A), 1.4 Å (holo L48Q), 1.9 Å (holo V44Q) and 1.2 Å (holo V79Q) was chosen, respectively. These cutoffs resulted in 3 (holo E40A, holo L48Q, apo V44Q), 4 (holo V44Q, apo E40A, apo L48Q, apo V79Q) and 2 (holo V79Q) clusters that represented at least 90% of the respective trajectories. Helical content for residues 54 through 68 was computed using dssp [38] and reported in units of amino-acid length. Interhelical angles between HA and HB were calculated using interhlx [39]. APBS [40] was used to compute the electrostatic potential for the proteins in openDX format. An ionic strength of 150 mM was assumed. Backbone N-H order parameters were calculated from the MD simulations of all apo and holo systems applying the isotropic reorientational eigenmode dynamics (iRED) approach [41] using a 0.5 ns window for averaging. The order parameters are calculated with the mat2s2.py script using a list of eigenvalues and eigenvectors of all the N-H backbone vectors generated by ptraj.

### Brownian dynamics simulations of diffusional-encounter association rates

Association rates for the diffusional encounter, 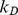 were computed using BrownDye [42]. PQR files of the cluster centers were generated using pdb2pqr [43]. The calcium pqr file was generated using a charge of +2 and an ionic radius of 1.14 Å. Bd_top was used to generate all necessary input files for the BrownDye runs. A phantom atom of zero charge and negative radius (−1.14 Å) was introduced after the first execution of bd_top. The phantom atom was placed at the position of the calcium ion from the trajectory frame. Its sole purpose is to define a reaction criterion that is spherically symmetric around the expected binding position of the calcium. The reaction criterion was chosen to be 3.5 Å within the calcium binding site. 500,000 single trajectory simulations were performed on 8 parallel processors using nam_simulation. The reaction rate constants were calculated using compute_rate_constant from the BrownDye package. A weighted average of the rate constants of each cluster center serves as an estimate of the overall association rate for the system.

## Supporting Information

Figure S1
**Histogram of distances between D67** 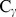 **and E76** 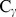 **of the apo-state structures.** Distances are reported in [Å] for E40A (black) V44Q (red), L48Q (green), and V79Q (blue).(TIF)

Figure S2
**Holo versus wild-type RMSD.** a) all b) 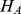, c) 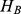, d) 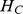, e) 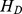 and f) 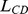 for E40A (black) V44Q (red), L48Q (green) V79Q (blue).(TIF)

Figure S3
**Distances between** 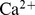 **and site II coordination residues.** a) D65 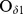 b) D65 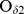, c) D67 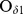 d) D67 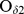 e) E76 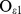 f) E76/for E40A (black) V44Q (red), L48Q (green) V79Q (blue).(TIF)

Figure S4 **Electrostatic potential of cTnC apo states.** Red and blue surfaces correspond to the −2.0 kT/e and 2.0 kT/e isopotential, respectively.
(TIF)

Figure S5
**cTnC V44Q compared against wild-type TnC holo and TnC-TnI bound states.** Representative holo V44Q structure (cyan) overlaid onto wild-type TnC with 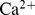 bound (gray) and TnI bound (caramel). TnI switch peptide fragment is in purple.(TIF)

Figure S6 **Comparison of wild-type and mutant helices adjacent to mutation site.** Ribbon representations of WT (cyan) versus a) holo E40A (black) b) holo V44Q (red) c) apo L48Q (green) and d) apo V79Q (blue). wild-type residues are colored by element type.
(TIF)

Video S1

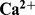
 **binding event.** Molecular dynamics trajectory depicting the binding of 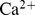 to wild-type troponin C.(MPG)
