# Supplementary figures and images for: Molecular Basis of Calcium-Sensitizing and Desensitizing Mutations of the Human Cardiac Troponin C Regulatory Domain: A Multi-Scale Simulation Study
Source: PLoS Comput Biol. 2012 Nov 29;8(11):e1002777. doi: 10.1371/journal.pcbi.1002777 (PMC3510055; doi:10.1371/journal.pcbi.1002777)

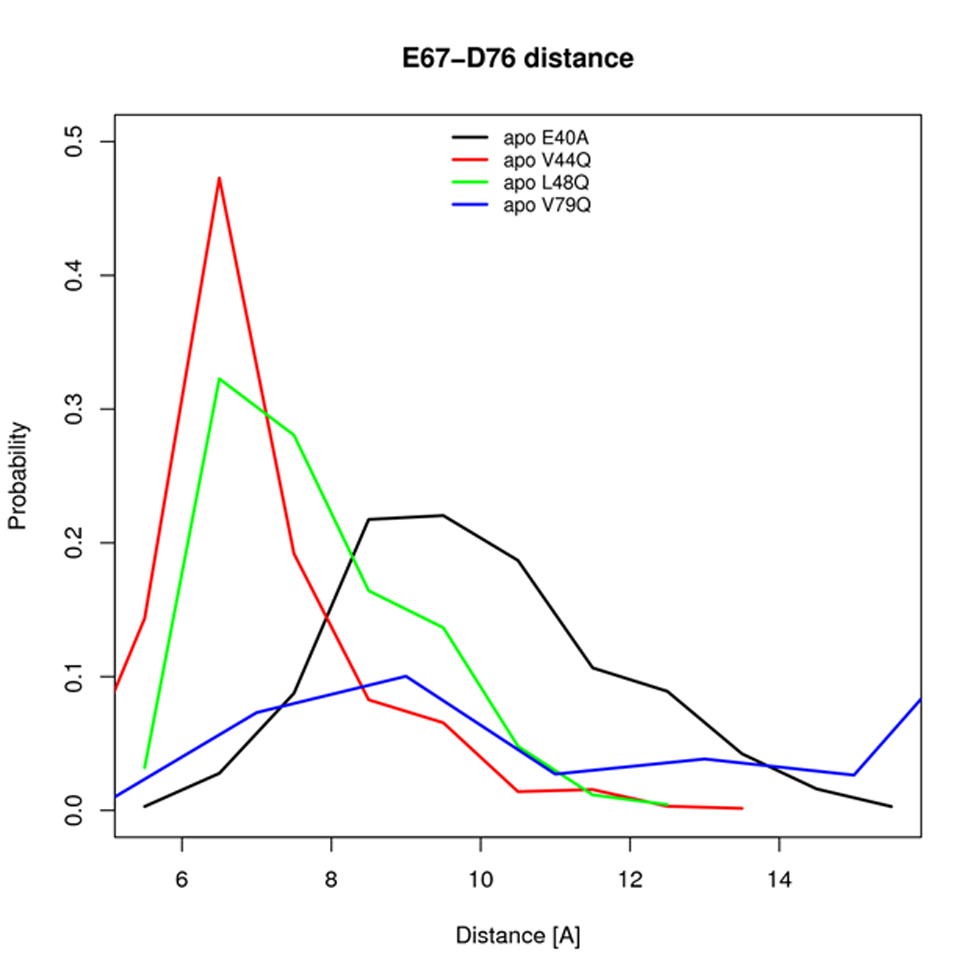

Supplement: Figure S1 — Histogram of distances between D67 and E76 of the apo-state structures. Distances are reported in [Å] for E40A (black) V44Q (red), L48Q (green), and V79Q (blue). (TIF) [file pcbi.1002777.s001.tif]

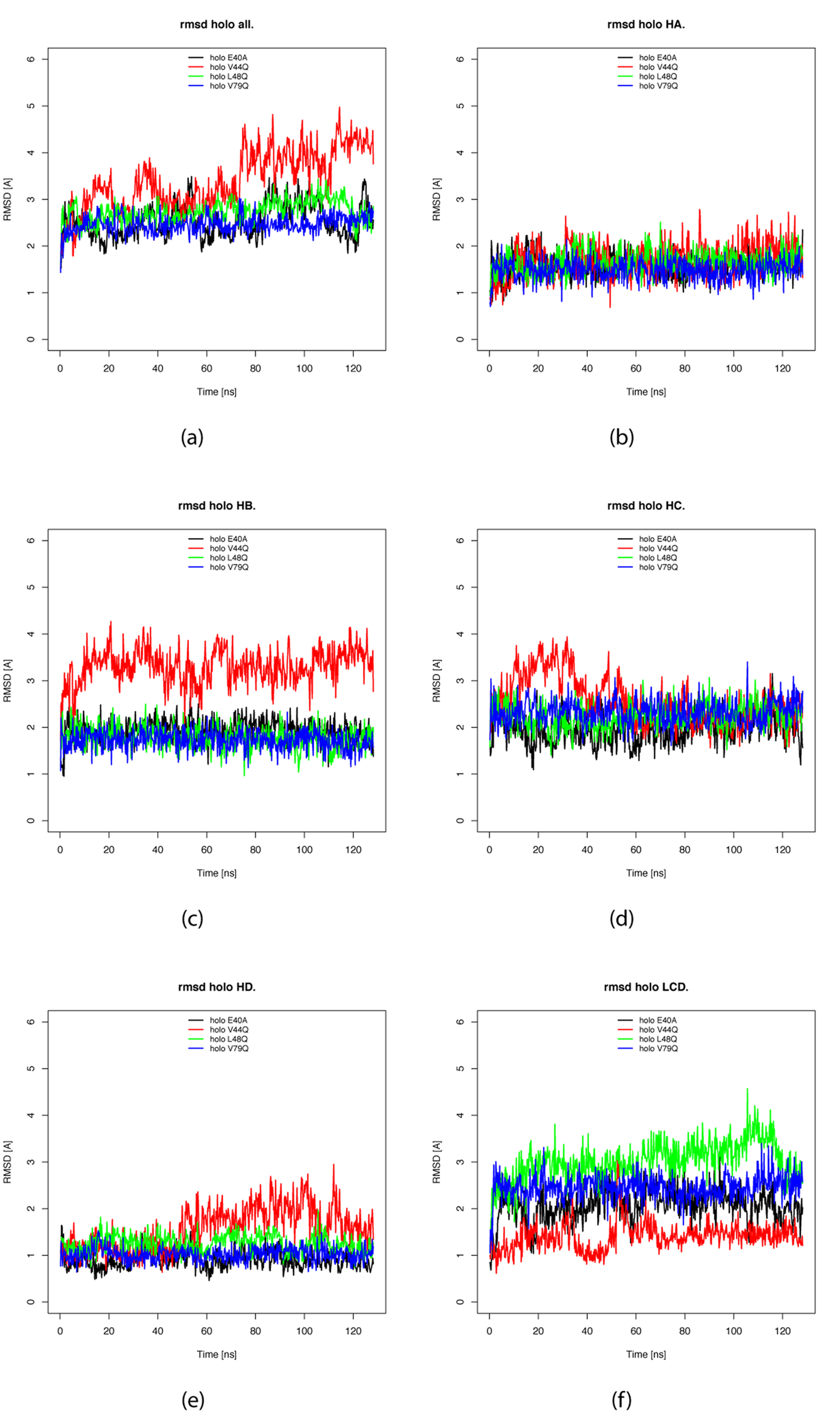

Supplement: Figure S2 — Holo versus wild-type RMSD. a) all b) , c) , d) , e) and f) for E40A (black) V44Q (red), L48Q (green) V79Q (blue). (TIF) [file pcbi.1002777.s002.tif]

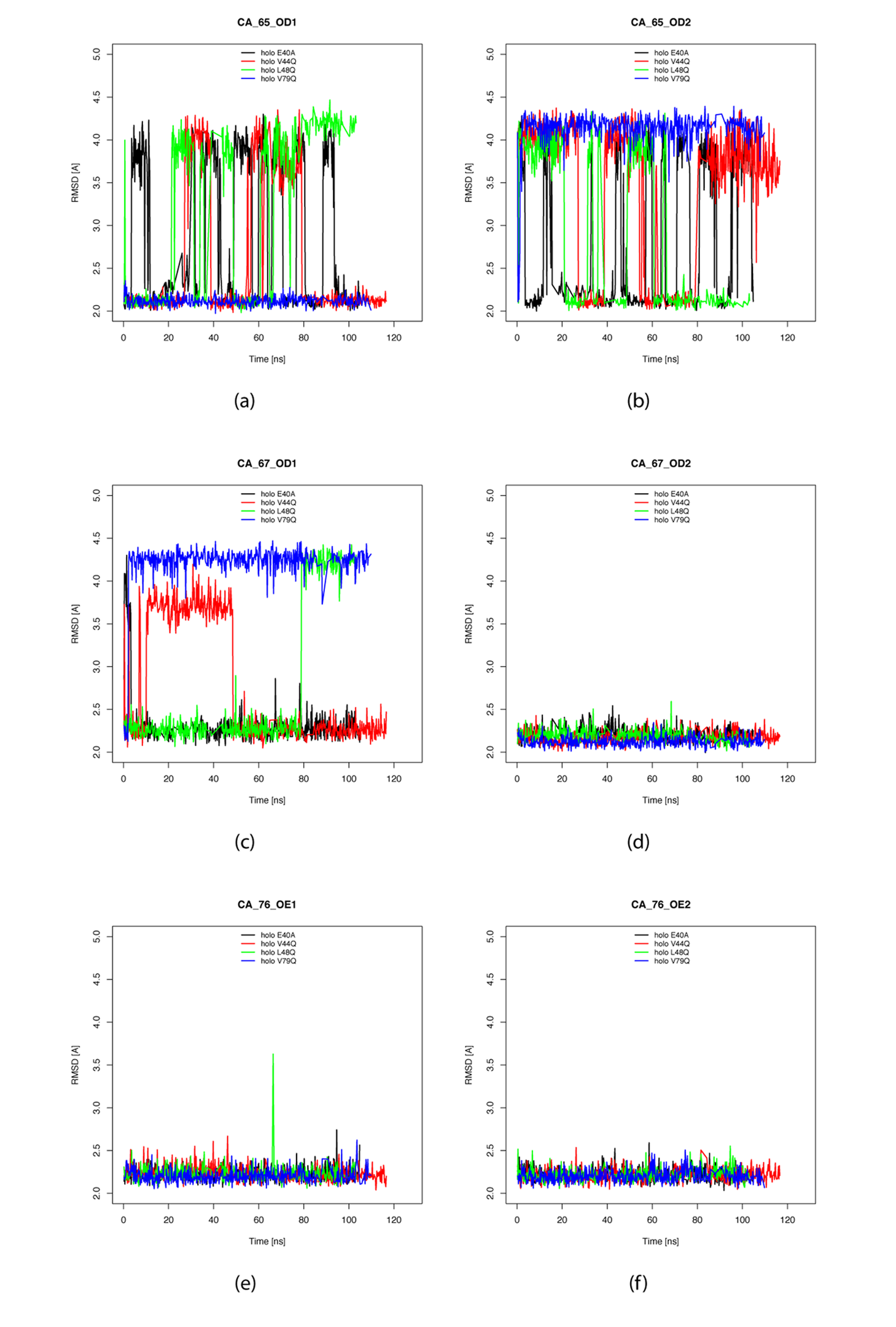

Supplement: Figure S3 — Distances between and site II coordination residues. a) D65 b) D65 , c) D67 d) D67 e) E76 f) E76/for E40A (black) V44Q (red), L48Q (green) V79Q (blue). (TIF) [file pcbi.1002777.s003.tif]

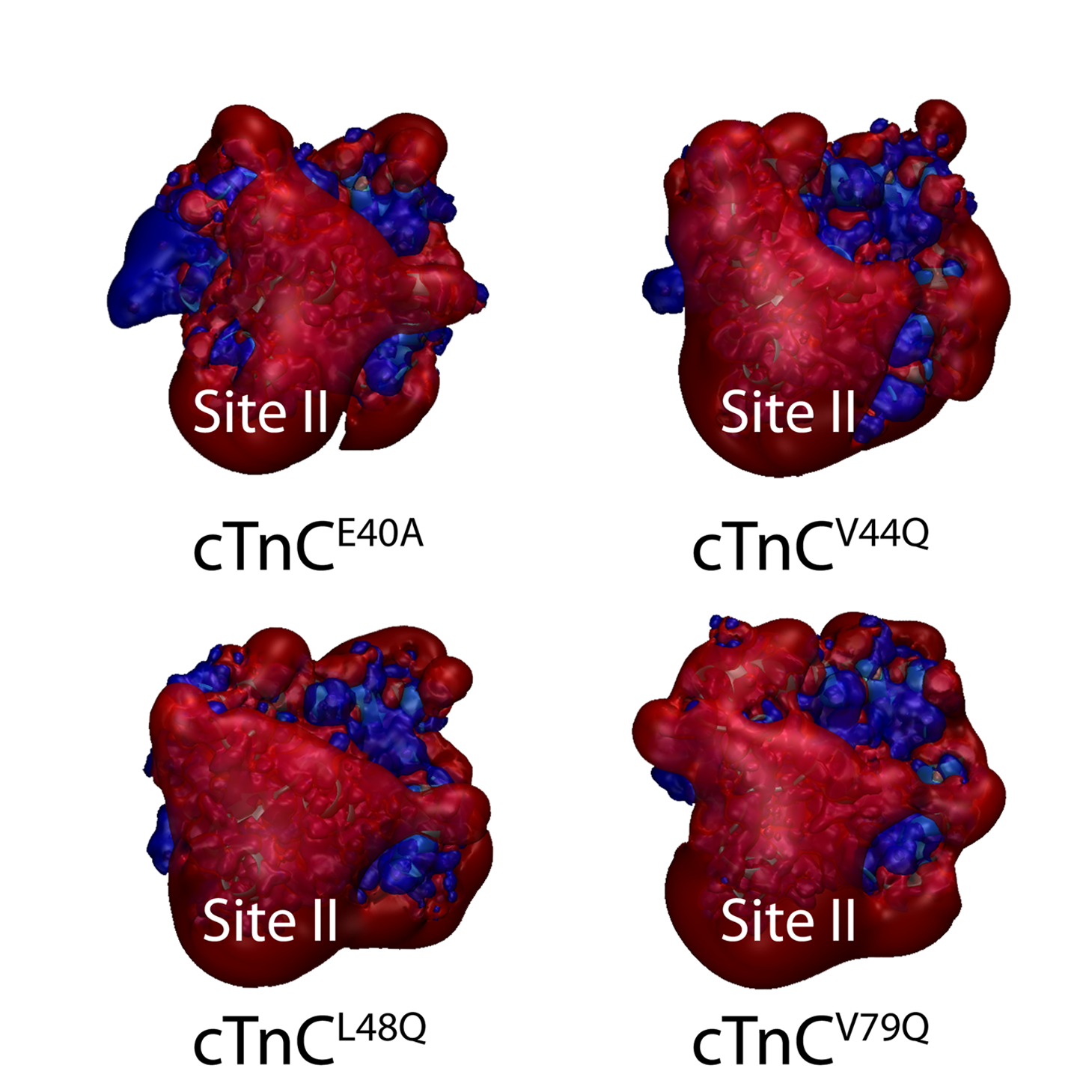

Supplement: Figure S4 — Electrostatic potential of cTnC apo states. Red and blue surfaces correspond to the −2.0 kT/e and 2.0 kT/e isopotential, respectively. (TIF) [file pcbi.1002777.s004.tif]

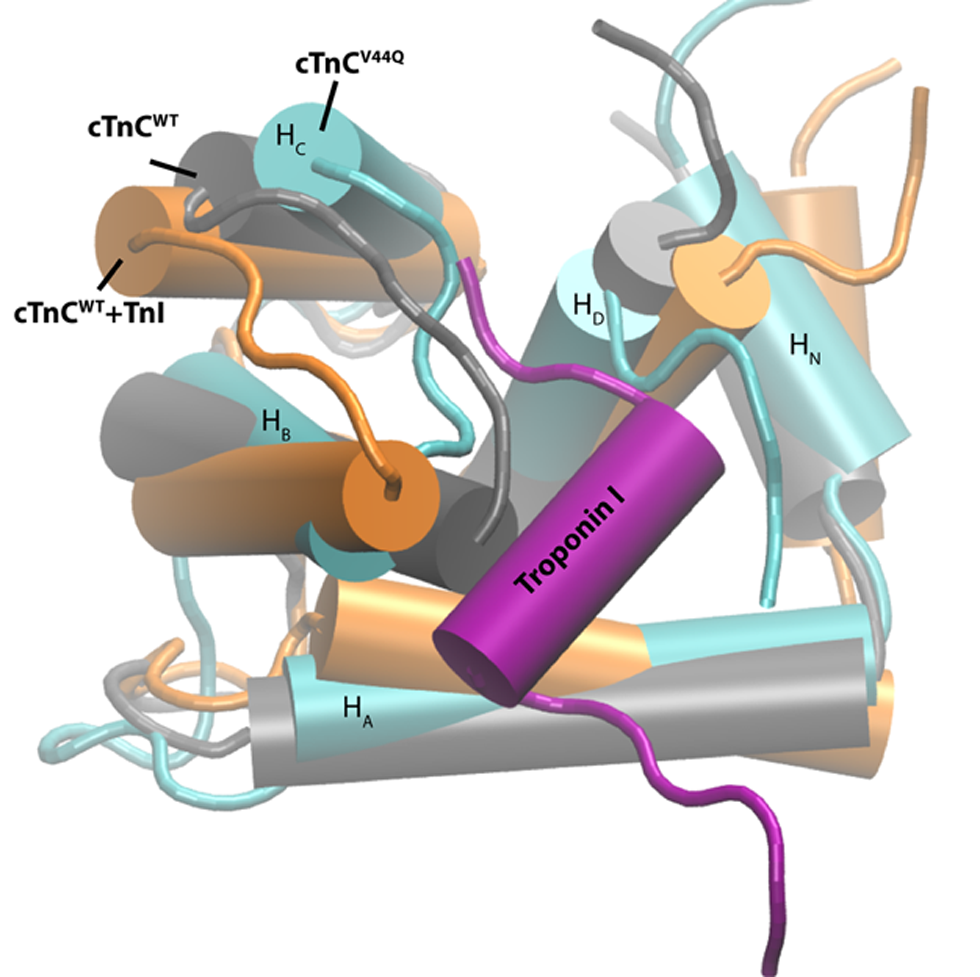

Supplement: Figure S5 — cTnC V44Q compared against wild-type TnC holo and TnC-TnI bound states. Representative holo V44Q structure (cyan) overlaid onto wild-type TnC with bound (gray) and TnI bound (caramel). TnI switch peptide fragment is in purple. (TIF) [file pcbi.1002777.s005.tif]

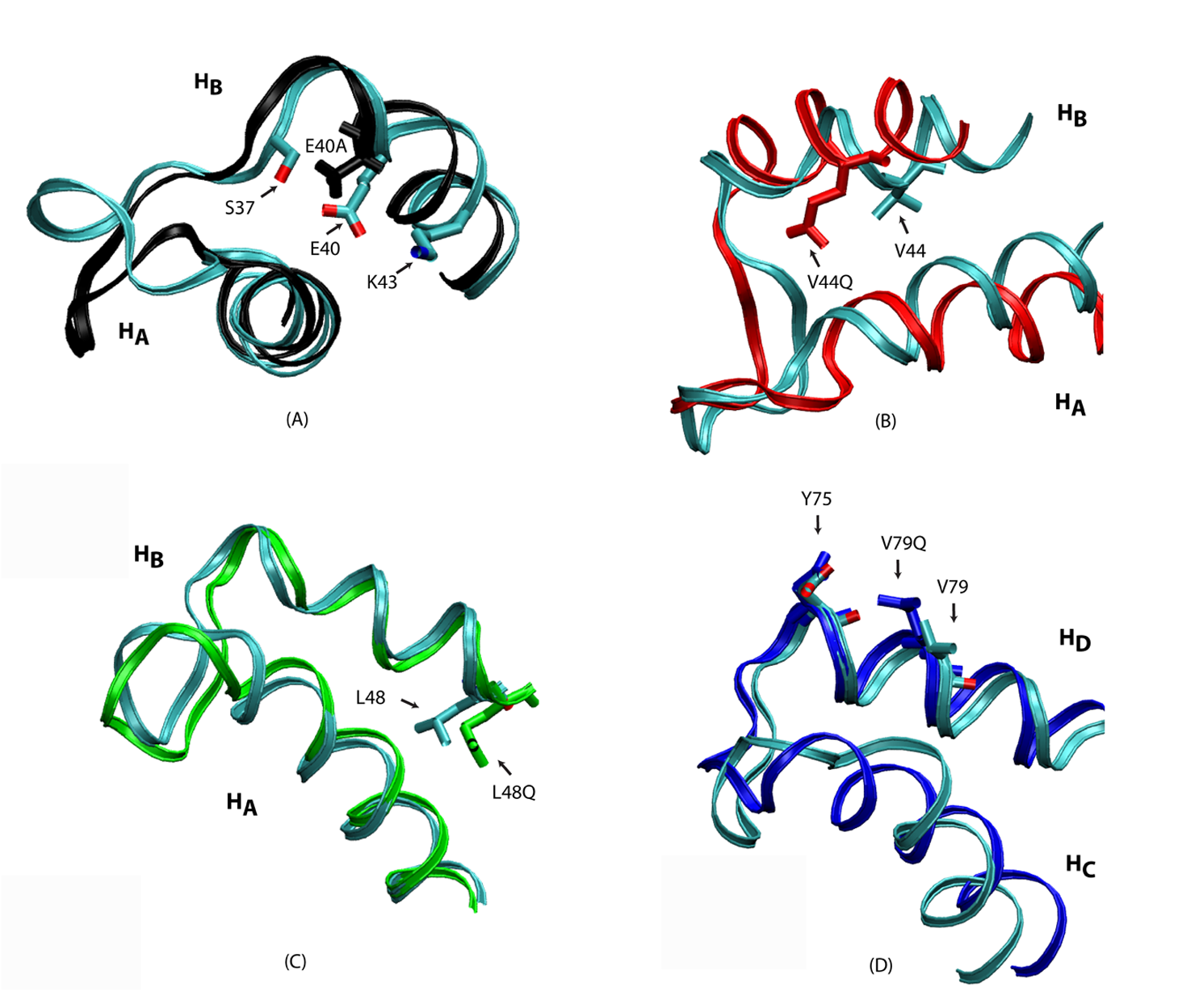

Supplement: Figure S6 — Comparison of wild-type and mutant helices adjacent to mutation site. Ribbon representations of WT (cyan) versus a) holo E40A (black) b) holo V44Q (red) c) apo L48Q (green) and d) apo V79Q (blue). wild-type residues are colored by element type. (TIF) [file pcbi.1002777.s006.tif]
